# The prevalence and economic burden of pain on middle-aged and elderly Chinese people: results from the China health and retirement longitudinal study

**DOI:** 10.1186/s12913-020-05461-6

**Published:** 2020-07-01

**Authors:** Yudian Qiu, Hu Li, Ziyi Yang, Qiang Liu, Kai Wang, Rujun Li, Dan Xing, Yunfei Hou, Jianhao Lin

**Affiliations:** Arthritis Clinic and Research Center, Peking University People’s Hospital, Beijing, China

**Keywords:** Pain, Prevalence, Economic burden

## Abstract

**Background:**

To estimate the prevalence of pain among people aged 45 years and older in China, to analyze the effect factors of pain and pain related economic burden.

**Methods:**

Nationally representative sample was derived from China Health and Retirement Longitudinal Study (CHARLS). Pain data, medical cost data were obtained, as well as information of demographic characteristics, social structure, social-economic status, other health needs and health behaviors. The prevalence of pain in 2011, 2013, and 2015 was calculated. Univariate analysis and multivariate analysis were used to find the effect factors of pain. An optimization two-part model was used to calculate the range of the direct medical costs caused by pain.

**Results:**

The prevalence of pain among people 45 years or older in China was 31.73% in 2011, 37.27% in 2013 and 28.62% in 2015. When evaluating factors lead a higher prevalence of pain, the results of the multi-variable after one-way analysis were older age, female, lower education, rural residents, without insurance status, abstained from alcohol and lower body mass index (BMI). Through the optimization of two-part model, the direct medical costs caused by pain was 898.9–1563.0 yuan in 2011, 2035.8–2568.7 yuan in 2013 and 2628.8–3945.7 yuan in 2015 (129.9US$ - 225.9US$ in 2011, 294.2 US$ - 371.2US$ in 2013 and 379.9US$ - 570.2US$ in 2015, converted to 2010 RMB).

**Conclusion:**

The prevalence of pain among middle-aged and elderly Chinese is high. Residents with older age, female, lower education, rural residents, without insurance status, abstained from alcohol and lower BMI seem to have a higher pain prevalence. Pain can cause extra direct medical costs and will cause more economic loss with the progress of time. Future research should pay more attention to effective treatment, management and prevention of pain to decrease its burden.

## Background

Pain is a common symptom which brings heavy burden to people’s life. It not only affects people’s overall health [1], but also seriously interferes with people’s daily activities, leading to depression, reduced social interaction and reduced quality of life [2]. There are many reasons and factors causing older adults’ status of pain, such as age, chronic disease status or unhealthy lifestyle. In the United States, the overall prevalence of pain is about 20.4% [3]. Among them, pain seems to have a greater impact on middle-aged and elderly people [4, 5]. It can be seen that with the progress of time and the aging of the global population, pain will become a more and more serious health problem. In order to study the disease burden associated with non-fatal diseases, the Global Burden of Disease Research (GBD) used the concept of disability, which is defined as “any short-term or long-term health loss”, and proposed “Disability Adjustment Life Year (DALY)” and “Years Lived with Disability (YLD)” as calculation indexes [6]. Between 1990 and 2010, among the 10 major causes of YLD, there were 5 diseases characterized by pain. In the global burden of disease study in 2016 [7], among the 5 major causes of YLD, low back pain and migraine ranked first and second place. Alexander K. Smith etc. mentioned in the study that in the last two years of life, the incidence of pain increased dramatically from 26 to 46% [8], regardless of the cause of death. Thus, pain can cause a lot of additional medical costs and economic losses over time, the burden of pain cannot be ignored.

China is a large country, with a population of over 1.3 billion. Over the past few decades, China has experienced tremendous development, including urbanization, income growth and aging, leading to a rapid increase in non-communicable diseases and a shift to chronic disability [9, 10]. In 2010, 25.3% of the population comprised individuals age 50 years or older in China [11]. Numerous studies have discussed the prevalence and economic burden of chronic pain in developed western nations, but for Chinese population, there are not many studies focusing on this area, most studies are about the mechanism and the treatment measures of pain. Studies have reported that rates of chronic pain in Chongqing appear to approximate to those reported in western countries [12]. A cross sectional study estimated the prevalence of pain and identify risk factors of pain among 19,665 community residents in China and found that Women had a higher prevalence of pain than men (39.9% vs. 32.2% for chronic pain) [13]. A cross-sectional study among 6524 elderly individuals aged ≥60 years in China reported the prevalence of chronic pain was 49.8%. The legs/feet (25.5%), back (23.2%), and neck/shoulder (14.6%) were the most salient locations for chronic pain, subjects with overweight and obesity were more likely to have chronic pain [14]. However, high quality data regarding the prevalence of pain and its health economic burden in China based on a large sample size is still needed [15]. Using data collected from the China Health and Retirement Longitudinal Study (CHARLS), a national random sample of the Chinese population, we estimated the prevalence of pain among residents age 45 years or older in China in the year 2011, 2013 and 2015 through this nationwide representative sample of the follow-up survey on health and pension. Also, we analyzed the effect factors for pain, and calculated the direct medical costs for all types of pain by using the optimized two-part estimation method.

## Methods

### Study population

CHARLS is a nationally representative longitudinal survey of the middle-aged and elderly population of China. Residents age 45 years and older and their spouses were interviewed at their homes, including assessments of the social, economic, and health status. A detailed description of the CHARLS was published previously [16].

**Fig. 1:**
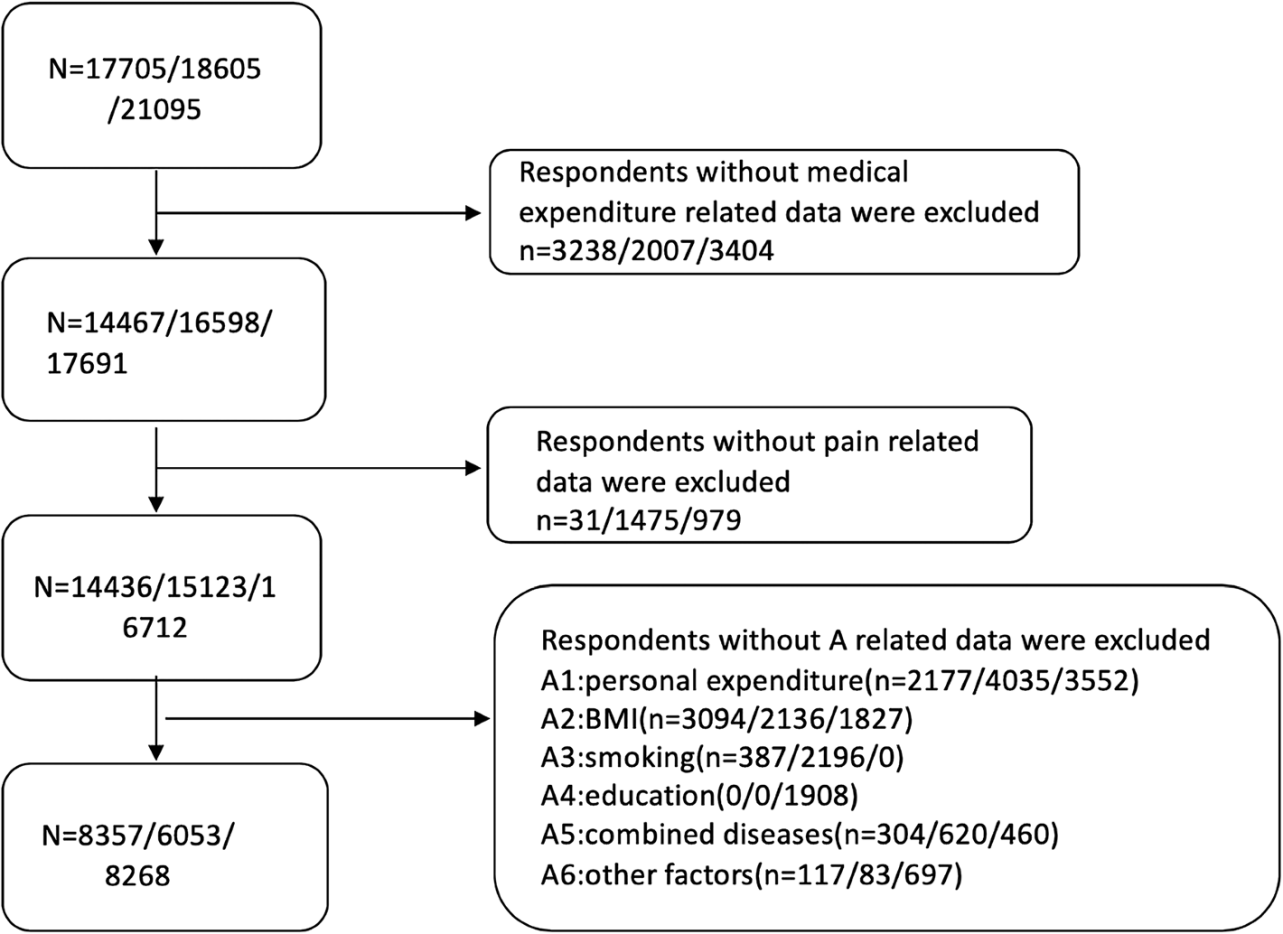
Data screening process (statistics in 2011/2013/2015)

Generalized, multistage probability sampling strategy and probability-proportional-to-size (PPS) sampling technique were applied in the baseline survey which was conducted between June 2011 and March 2012. Four stages of sampling procedures (county-level sampling, neighborhood-level sampling, household-level sampling and respondent-level sampling) were used to obtain a national representative sample [16]. In the sampling stage at county-level, 150 counties are randomly selected from 30 provincial administrative units (excluding Tibet Autonomous Region, Taiwan Province, Hong Kong and Macao Special Administrative Regions) in China according to the PPS method, based on the population of each district and county in 2009, using the region, urban and rural areas and GDP as hierarchical indicators. In the sampling stage at village level sampling stage, according to the PPS method, based on the 2009 resident population of each village or community, three villages were randomly selected from each of the above 150 districts and counties, and finally 450 villages were obtained. The above sampling process is carried out in Stata software environment, and it is not allowed to change samples. In order to avoid the deviation of population information, we compared the resident population data of 450 village units in 2009 with that in 2007. For villages where the difference in population data over two years exceeds a certain limit, verification has been made to the Bureau of statistics. At the same time, for the selected villages, the quality of the sampling was further guaranteed through the document issued by the CDC to the whole country for verification. The final sample of 450 administrative villages and neighborhoods in 150 counties were selected, including over 17,000 individual participants. CHARLS respondents were followed in 2013 and 2015 through a face-to-face computer-assisted personal interview (CAPI), including follow-up of previous respondents and survey of new participants. After data screening, 8357, 6053 and 8268 respondents met the research requirements in 2011, 2013 and 2015, respectively (see in Fig. 1).

### Data collection

Information collected during the household interview included demographic characteristics (i.e., age, gender, residence address), social structure (marital status, job availability), socioeconomic status (i.e., education, per capita expenditure (PCE)), and medical history (chronic diseases). Data on health behaviors (such as smoking, alcohol consumption, obesity, body mass index (BMI)) were also collected to minimize the impact of confounding factors and to obtain the correlation between pain and medical costs.

Participants were first asked whether they often “troubled” any physical pain. We used data from the CHARLS to study the direct medical costs of pain. The medical cost was self-reported as the total amount paid during one month (for outpatient and self-treatment) or one year (for inpatient) preceding the survey date. We classified healthcare expenditure into three types: outpatient expenditure, inpatient expenditure, and self-treatment expenditure. Outpatient expenditure in the past month was calculated as the sum of reported expenditure for visiting a general hospital, specialized hospital, Chinese medicine hospital, community healthcare center, township hospital, health care post, Village clinic/Private clinic, including both treatment and prescriptions the respondents received, during the one month preceding the survey date. Inpatient expenditure in the past year was calculated as the sum of all the reported fees paid to the hospital, including medication fees, surgery fees, ward fees, laboratory test fees, medical check-up/consultation fees and other fees, but excluding wages paid to a hired care worker, transportation expenditure, and accommodation costs for the respondent or his/her family, during the one year preceding the survey date. Self-treatment expenditure in the past month was calculated as the sum of reported expenditure for treatment without resorting to professional medical care, such as over-the-counter/prescription medicines, traditional herbs or traditional medicines as treatment, tonic/health supplements, and the use of healthcare equipment, during the one month preceding the survey date. In order to make the data from the three time point comparable, we converted the cost into RMB according to the consumer price index (CPI) in 2010. In this paper, the principle of data screening is to exclude those with missing values.

We categorized the ages of the subjects into 4 groups (45–54 years, 55–64 years, 65–74 years, and ≥ 70 years) and categorized their living education into 4 groups: no formal education, elementary school, middle/high school, and college degree or higher. All the participants were classified as an urban or rural resident. The insurance situation of the subjects was divided into 3 categories: no insurance, basic medical insurance and other types of insurance. The BMI was divided into four categories: “low weight” (< 18.5), “normal” (18.5–24.9), “overweight” (25–29.9) and “obesity” (≥ 30). We divided the smoking/drinking situation of the respondents into three categories: “presently smoking/drinking” represent “yes” for smoking/drinking status, “previous smoking /drinking and quit now” represent “abstained from smoking/drinking” for smoking/drinking status, “never smoked/drank” represent “no” for smoking/drinking status. In the study of household economic status, PCE were used for its stability. Per capita expenditure was trisected into three parts: low, middle and high expenditure groups.

We used CHARLS data to study the direct medical costs of pain. We collect the medical costs of outpatient, inpatient and self-treatment for each interviewee from the CHARLS dataset, excluding indirect medical costs and intangible medical costs such as charge for loss of working time. Smoking or not quitting smoking is judged as smoking status, and drinking more than once a month in the past year is judged as drinking status.

### Statistical analysis

We used the SPSS version 25.0 to calculate the population characteristics in 2011, 2013 and 2015, and calculated the prevalence of pain based on the data in the CHARLS study. Individual weight with household and individual non-response was incorporated in the analysis by STATA version 15.0 to estimate the prevalence of pain and the direct medical costs caused by pain among people 45 years or older in China.

To explore the effect factors of pain, we selected the baseline data of 2011. We performed univariate and multivariate analysis of pain distribution in different subgroups, using pearson chi-square test and logistic regression. We applied single factor and multi-factor analysis using one-way ANOVA, Brown-Forsythe test and Binary logistic regression. The criterion of significance is *p* <  0.05.

The “two-part model of the demand for medical care” was used in our study to estimate the direct medical cost per capita of people with pain symptoms [17]. The first part is probability prediction model (a logistic regression model), which estimates the probability of generating medical costs (we set the medical cost as a categorized variable of “yes” and “no”). And the second part of the model is a generalized linear model with logarithmic gamma distribution, which is used to estimate the direct medical costs of respondents whose medical costs are positive. The expected value of medical cost is calculated by multiplying the probability obtained in the first part by the predicted costs obtained in the second part. The direct medical cost of pain is then counted by calculating the difference between the expected medical cost of pain and non-pain population. Considering the uncertainty of the direction and extent of the impact of confounding factors on the direct medical costs caused by pain. Two models were used to complete the analysis. In model 1, only demographic characteristics and individual family capacity were included as independent variables besides pain. In model 2, other health needs and health behaviors were included on the basis of model 1 as independent variables. The two models together were used to estimate the range of additional direct medical costs caused by pain.

## Results

After data screening, 8357, 6053 and 8268 respondents met the research requirements in 2011, 2013 and 2015 respectively. As shown in Table 1, the majority of the participants were women, primary schools education level, subjects lived in rural areas, aged between 45 and 64, the coverage rate of resident insurance in China is about 90%. As shown in Table 2, the overall prevalence of pain in CHARLS study was 30.41% in 2011, 37.62% in 2013 and 27.56% in 2015(The main parts were waist, leg and knee). After individual weight was incorporated, the prevalence of pain among people 45 years or older in China was 31.73% in 2011, 37.27% in 2013 and 28.62% in 2015.

**Table 1:** Descriptive characteristics of respondents in CHARLS study

year	2011	2013	2015
total residents, n	8357	6053	8268
age, n (%)			
45–54	32.68	34.63	30.95
55–64	38.64	36.40	34.83
65–74	20.26	21.99	25.48
> =75	8.42	6.99	8.73
gender, n (%)			
male	48.07	35.65	47.80
female	51.93	64.35	52.20
education level, n (%)			
no formal education	27.01	27.36	23.43
elementary school	61.86	61.72	64.71
middle/high school	9.76	9.28	9.99
college degree or higher	1.36	1.64	1.87
origin, n (%)			
urban	35.83	35.29	36.02
rural	64.17	64.71	63.98
insurance status, n (%)			
no insurance	6.55	3.67	10.28
basic medical insurance	92.45	95.80	85.63
other types	1.01	0.53	4.09
smoking status, n (%)			
yes	31.24	18.16	29.70
no or abstained from smoking	68.76	81.84	70.30
drinking status, n (%)			
yes	25.67	21.76	27.54
no or abstained from drinking	74.33	78.24	72.46
BMI, n (%)			
low weight	6.62	5.01	5.31
normal	62.67	56.22	59.40
overweight	25.79	31.95	29.96
obesity	4.93	6.82	5.33

**Table 2:** The prevalence and location of pain in CHARLS study

year	2011	2013	2015
pain status, n (%)			
yes	30.41	37.62	27.56
no	69.59	62.38	72.44
the location of pain, n (%)			
head	11.81	9.27	13.13
shoulder	11.38	9.18	13.24
arm	9.00	7.19	10.53
wrist	5.60	4.05	7.67
finger	5.28	4.86	7.76
chest	5.58	4.53	5.54
stomach	7.63	4.51	8.93
back	8.34	7.07	9.98
waist	17.98	16.44	18.24
hip	3.39	2.46	5.35
leg	13.40	12.29	13.87
knee	12.31	9.66	14.26
ankle	5.18	4.71	7.10
toes	2.90	2.63	4.60
neck	5.82	4.81	8.49

Distribution of pain in different populations, univariate and multivariate analysis results are shown in Table 3. We found that the prevalence of pain was higher in the population with the following risk factors: older age, female, lower BMI, lower education level, lower expenditure level, rural residents, no insurance, non-smoker, non-alcoholism and abstained from alcohol. After the multivariate analysis, the three previous risk factors: lower expenditure level, non-smoker and non-alcoholism were excluded. The rest factors may be independent risk factors for pain. The results of single factor analysis as well as multivariate analysis of direct medical costs all showed that pain have a positive impact on direct medical costs (*P* <  0.05).

**Table 3:** Univariate and multivariate analysis results of risk factors for pain

		univariate analysis		multivariate analysis	
		χ2	*p* value	OR (95%CI)	*p* value
age					
45–54	0.2761	17.04	0.002		
55–64	0.3159			1.17 (1.04,1.32)	0.007
65–74	0.3284			1.23 (1.08,1.42)	0.003
> =75	0.2997			1.03 (0.84,1.26)	0.750
gender					
male	0.2549	88.27	< 0.001		
female	0.3495			1.51 (1.36,1.68)	< 0.001
education level					
no formal education	0.37	125.17	< 0.001		
elementary school	0.2988			0.90 (0.80,1.01)	0.080
middle/high school	0.1826			0.55 (0.44,0.68)	< 0.001
college degree or higher	0.1053			0.34 (0.18,0.63)	0.001
marriage					
yes	0.301	2.42	0.12		
no	0.3242			1.05 (0.90,1.22)	0.508
per capita expenditure					
low	0.3156	14.25	0.001		
middle	0.3179			1.11 (0.99,1.25)	0.072
high	0.2757			1.05 (0.92,1.19)	0.438
working status					
yes	0.304	< 0.01	0.982		
no	0.3042			1.03 (0.91,1.16)	0.602
origin					
rural	0.342	101.70	< 0.001		
urban	0.2361			0.64 (0.57,0.70)	< 0.001
insurance status					
no insurance	0.3601	17.18	< 0.001		
basic medical insurance	0.3017			0.73 (0.61,0.88)	0.001
other types	0.1548			0.42 (0.23,0.79)	0.007
smoking status					
no	0.3209	17.26	< 0.001		
abstained from smoking	0.2879			1.20 (0.98,1.46)	0.074
yes	0.2761			1.15 (0.99,1.32)	0.062
drinking status					
no	0.3141	14.88	0.001		
abstained from alcohol	0.3284			1.34 (1.07,1.65)	0.009
yes	0.2713			1.06 (0.93,1.21)	0.355
BMI					
normal	0.296	12.94	0.005		
low weight	0.3689			1.25 (1.03,1.51)	0.023
overweight	0.3086			1.11 (0.98,1.23)	0.094
obesity	0.2961			1.00 (0.79,1.25)	0.980

**Fig. 2:**
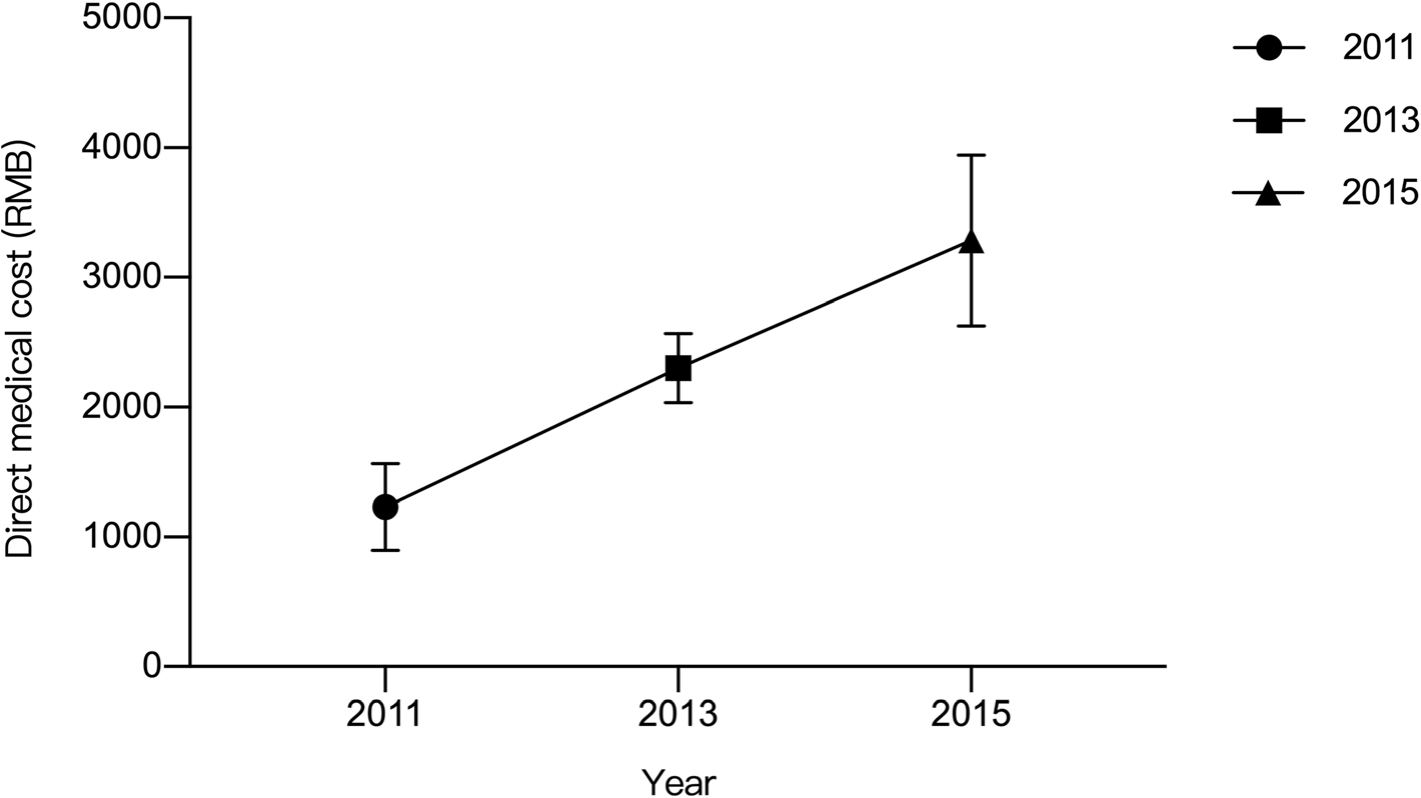
Direct medical cost caused by pain in two-part model

By calculating the difference of medical cost expectation between pain and non-pain population through the optimization of two-part model, we got the direct medical cost caused by pain: 898.9–1563.0 yuan in 2011, 2035.8–2568.7 yuan in 2013 and 2628.8–3945.7 yuan in 2015 (129.9US$ - 225.9US$ in 2011, 294.2 US$ - 371.2US$ in 2013 and 379.9US$ - 570.2US$ in 2015, converted to 2010 RMB, see in Fig. 2). The direct medical costs caused by pain are mainly concentrated in outpatient clinics, the results of three surveys show that the additional direct medical costs in outpatient clinics are about 50% of the total.

## Discussion

Using data collected from the CHARLS, a national population survey, we observed that pain symptoms was common among Chinese adults, especially among those with lower socioeconomic status. There was remarkable variation in the prevalence of pain according to socioeconomic status. Subjects with older age, female, low weight, lower education level, rural residents, no insurance and abstained from alcohol seems to have much higher prevalence.

The prevalence of pain in our study was significantly higher than that in Japan (17.5%) [18], France (20.2%) [19], the United States (20.4%) [3] and other countries [1, 3, 18–20]. In a systematic review, the prevalence of pain ranged from 0 to 24.0%worldwide [21]. A report from China National Committee on Ageing have shown approximately 30.9% of the urban older adults and 38.7% of the rural older adults were reported to have pain [22], which are quite close to our analysis in this study. The high prevalence of pain in this study may be related to the following two reasons. First of all, the age of the population in our study was ≥45, while older age was a related risk factor for pain [23–26], the U.S. and France studied the whole population, while Germany was the population over 14 years old. Secondly, the relatively low economic level of the population investigated in this study may also be one of the reasons. Low income was considered as a risk factor for pain in some studies [25]. A multivariate analysis of pain-related factors was conducted showing that there was a correlation between economic conditions and pain intensity as well as the consequences of pain. In this study, the proportion of rural residents accounted for the majority, which may lead to inaccuracy of our estimation.

From the results of our study, we can tell the proportion of pain caused by musculoskeletal diseases is the highest, in which low back pain take the first seat in all parts of the body. Leg, knee, head, shoulder, and arm are the common pain areas with a prevalence of more than 10%. Except for the head, other part all belong to the musculoskeletal system. In the global burden of disease study in 2016, low back pain ranked first among the five major causes of YLD [7]. It can be seen that the high prevalence of low back pain is one of the reasons for its high health burden. Some studies have shown that the prevalence of low back pain in adults ranges from 14.7 to 23.6% [26–28], which is consistent with our results. It can be seen that musculoskeletal diseases play an important role in both pain and YLD, which need to be paid more attention to.

Our study suggest that older age and female are independent risk factors for pain, which is consistent with some previous research results. A study of Swedish residents showed that the prevalence of chronic musculoskeletal pain increased with aging, and reached the highest between the ages of 59 and 74 [23]. In our study, the highest prevalence of pain was found in the age group of 65–74, which is close to the results of the previous study [29, 30]. Aging showed a positive correlation with increased incidence of cancer, osteoarthritis, spinal diseases, surgical injuries and other diseases, which may lead to pain prevalence [31]. Studies have found that the prevalence of pain, and musculoskeletal diseases in women was higher than that in men [14, 32]. One of the most common opinions on gender differences in pain is that women are more sensitive to pain. An electrical stimulation study found that the amplitude of female evoked potential was higher than that of male [33]. Hormones may play a role [34]. In addition, some scholars believe that gender differences in pain are related to psychological factors. For example, men are more reluctant to report pain than women [35].

In addition to demographic characteristics, we also studied the relationship between social structure and pain. Combining the results of univariate and multivariate analysis. We found a negative correlation between education and pain, which is consistent with some studies [36, 37], suggesting that higher education level may accomplished by higher pain endurance. In this study, we also found that the prevalence of pain among rural residents is higher, which is consistent with the results of the study on farmers in Latin America and women in Tibet [38, 39], subjects live in rural areas and, with lower education level seem to report their pain more easily. In terms of medical insurance, we found that the prevalence of pain in uninsured people is higher. People with low back pain in the United States had lower insurance coverage and were more likely to receive medical assistance [40]. The impact on pain may come from many aspects, such as the difficulty of access to health resources (including health-related education resources), medical concept and preference. In addition, the impact of different occupations is tremendous, some occupations may be more vulnerable to the threat of pain, especially manual workers. For example, the prevalence of low back pain in Chinese garment workers (74%) was significantly higher than that in teachers (40%) [41]. It is generally believed that manual workers may be more threatened by pain. However, low education level, rural residents usually engage in more manual labor, and manual workers have lower coverage of health insurance. Documents have proved that physical intensive work, represented by agriculture, is more common in rural areas than in urban areas [42]. In our country, there are more rural residents, and according to the CHARLS baseline study report, many rural residents in our country do not stop working until 65 years old, and at least 20% of people over 80 years old are still working. Therefore, pain should be paid more attention in our country.

In terms of health behavior, we found that abstinence from alcohol and low BMI may be independent risk factors for pain, while smoking is not, which differs from previous studies [43–45], different set of cut-points for BMI didn’t influence this result. The relationship between smoking and pain may be considered in two ways. On the one hand, a large number of studies have shown that smoking increases the incidence of pain in certain parts [43–45]. On the other hand, smoking may improve people’s bad mood, and the subjective factors of pain symptoms are strong, so smokers may tend not to report pain. Studies showed that nicotine can stimulate the release of dopamine to produce a relaxing and pleasant subjective experience, and nicotine also has an acute analgesic effect [46, 47]. Perhaps it is for these two reasons that we did not find a correlation between smoking and pain in our study. In terms of alcohol consumption, our conclusions differ from those commonly believed that alcohol consumption may lead to a higher incidence of pain. However, previous studies have shown that abstainers have a higher incidence of pain. For example, the prevalence of migraine tended to decrease with the increase of alcohol consumption compared with abstinence, and explained it as the deposition characteristics of alcohol [48]. In addition, the prevalence of pain among abstainers is higher, probably because many abstainers are passively abstaining from alcohol because of some illnesses, which in turn have painful symptoms. The relationship between low BMI and pain may be related to some diseases, and malnutrition is a risk factor for some chronic non-communicable diseases. Barbara et al. studied whether nutritional risk was associated with chronic musculoskeletal pain in the elderly living in the community by scales. The results showed that nutritional risk score was independently associated with chronic musculoskeletal pain. For each additional unit of risk score, the risk of pain increased by 11% [49].

Pain will cause more additional medical costs. According to the data of the National Bureau of Statistics, in 2011, 2013 and 2015, the total population of China was 1347.35 million, 1360.72 million and 1374.62 million [50]. It can be estimated that the economic losses caused by pain in China in 2011, 2013 and 2015 are 352.99–613.77 billion yuan, 1051.94–1327.29 billion yuan and 959.02–1439.45 billion yuan (converted to 2010 RMB), respectively. The cost of pain-related medical care in the United States in 2010 was $261 billion to $300 billion [51], which was 1728.52–1986.81 billion yuan. Generally speaking, although the additional direct medical cost caused by pain in China may be lower than that in some developed countries such as the United States, it will still cause greater economic losses.

The innovation of this study is to design two models to calculate the approximate range of additional direct medical costs caused by pain. Model 1 included fewer confounding factors, only the most important demographic characteristics and individual family competence characteristics. Therefore, the additional direct medical costs caused by pain may be underestimated. Health needs and health behaviors were incorporated in model 2, which may have a positive impact on the direct medical costs through pain. Therefore, when these factors are included as confounding factors, the additional direct medical costs of pain calculated by model 2 may be overestimated. In this way, we can estimate the range of additional direct medical costs caused by pain through the two models.

There’re also several limitations in our study. Firstly, the main disadvantage is the high missing value in the process of data screening, especially in 2013, which may lead to a certain selection bias, resulting in a decline in the credibility of the results. Secondly, this study lacks a specific scale for the collection of pain data, which is strongly effected by the subjectivity of the respondents. Thirdly, this study does not include the calculation of indirect medical costs caused by pain, so the estimation of economic burden caused by pain is conservative. Fourthly, the financial burden of pain may still be underestimated. On the one hand, we only include people aged 45 and over, but not under 45. On the other hand, our study did not calculate the additional indirect medical costs associated with pain, such as the cost of missed work, the cost of hiring a nanny, etc. However, due to less research on pain-induced economic burden and lack of overall data, the result in this paper is still irreplaceable and necessary. Further research is needed to start with different severity of pain and different types of medical costs, focusing on the collection of relevant data for deeper longitudinal research.

## Conclusions

The prevalence of pain is high among the middle-aged and elderly people in China, and many factors will lead to an increase in the prevalence of pain. Pain can cause more additional direct medical costs, and the costs are increasing over time. Therefore, we need to pay more attention to pain, conduct more in-depth research on pain, and strengthen pain related education for patients, especially those who are more likely to suffer from pain, in order to better treat, manage and prevent pain occurrence.

## Data Availability

Details of how to access the CHARLS data and details of the data release schedule are available from http://charls.pku.edu.cn/pages/data/111/zh-cn.html

## Abbreviations

CHARLS: China Health and Retirement Longitudinal Study
SD: Standard deviation
BMI: Body mass index
GBD: Global Burden of Disease Research
DALY: Disability Adjustment Life Year
YLD: Years Lived with Disability
PPS: Probability-proportional-to-size
CAPI: Computer-assisted personal interview
PCE: Per capita expenditure
CPI: Consumer price index
